# Bone Marrow Concentrate in the Treatment of Aneurysmal Bone Cysts: A Case Series Study

**DOI:** 10.1155/2020/8898145

**Published:** 2020-08-21

**Authors:** Lorenzo Andreani, Sheila Shytaj, Elisabetta Neri, Fabio Cosseddu, Antonio D'Arienzo, Rodolfo Capanna

**Affiliations:** Azienda Ospedaliera Universitaria Pisana, Via Paradisa 2, Pisa 56124, Italy

## Abstract

**Introduction:**

A recent attractive option regarding mesenchymal stem cells (MSC) application is the treatment of bone cystic lesions and in particular aneurysmal bone cysts (ABC), in order to stimulate intrinsic healing. We performed a retrospective evaluation of the results obtained at our institution.

**Methods:**

The study group consisted of 46 cases with an average follow-up of 33 months. Forty-two patients underwent percutaneous treatment as the first approach; four patients had curettage as first treatment. In all cases, autologous bone marrow concentrate (BMC) was associated too. The healing status was followed up through a plain radiograph 45 days and 2 months after the procedure. *Results and Conclusions.* At the final follow-up, thirty-six patients healed with a Neer type II aspect, nine healed with a type I aspect, and one patient was not classified having total hip arthroplasty. Bone marrow concentrate is easy to obtain and to manipulate and can be immediately available in a clinical setting. We can assert that the use of BMC must be encouraged being harmless and having an unquestionable high osteogenic and healing potential in bone defects.

## 1. Introduction

In the past 30 years, many studies have confirmed the potentials and plasticity of the so-called mesenchymal stem cells (MSC): they have been shown to reside within the connective tissues of most organs and can differentiate into osteogenic, adipogenic, and chondrogenic lineages under appropriate conditions [1–3]. These features have led to an increasing application of MSC in the orthopaedic field, especially when a strong regenerative capacity applied through a minimally invasive approaches is required. A simple method to have MSC is the bone marrow concentrate (BMC) obtained through autologous bone marrow aspiration and centrifugation. Local application of BMC is one of the current available treatments of bone defects and in particular of cystic lesions [4, 5]. Among them aneurysmal bone cysts (ABC) are uncommon osteolytic lesions, usually eccentric, with a hyperplastic behaviour often arising in the long bones of young people, being rare after 30 years of age [6]. The treatment approach to ABC has evolved in the past years ranging from radiotherapy to resection. More recently, less invasive procedures such as complete or partial curettage, and various substance injections seemed to be promising [7, 8]. We performed a retrospective evaluation of the results obtained at our institution after aneurysmal bone cyst percutaneous treatment with application of BMC in order to understand if the use of bone marrow stem cells actually gives benefits.

## 2. Material and Methods

We present a retrospective study involving 57 patients with diagnosis of aneurysmal bone cyst, treated at our institution between January 2013 and June 2019. The following exclusion criteria were applied: secondary ABC, initial treatment in other institution, and incomplete radiographic evaluation. Eight patients were excluded according to the following criteria: 2 ABC associated to chondroblastoma, 3 patient who had initial treatment in another hospital, and 6 patients for the absence of complete radiographic evaluation available. The study group consisted of 46 patients with an average follow-up of 33 months. Forty-two patients underwent percutaneous treatment as first approach; four patients had curettage as first treatment. The patients enrolled performed a plain radiograph before intervention, and the following radiographic variables were assessed: affected bone, staging according to the Enneking system [9], and location according to the Capanna classification [10]. In all cases, the diagnosis was obtained through a histopathological study of specimens collected intraoperatively. All of the procedures were performed using sedation and local anaesthesia or under general anaesthesia with an aseptic technique. The percutaneous injections were performed introducing a Jamshidi needle, under fluoroscopic guide, into the lesion. The content of the cyst was then aspirated and sent for histopathologic evaluation; subsequently, the chosen drugs were injected into the lesion. The injected substances were : vitamin C, atossisclerol (0, 25%), methylprednisolone acetate, and autologous bone marrow concentrate (BMC). The two lesions treated with curettage were filled with bone chips enriched with the same substances aforementioned. The BMC was obtained through aspiration of bone marrow from the ipsilateral iliac crest and centrifugation in a dedicated RegenKit® device. The healing status was followed up through a plain radiograph 45 days after the procedure and then 2 months after. According to the modified Neer classification [11], the procedure was repeated if no satisfactory healing of the lesion occurred after 2 months, i.e. if the radiolucent area was ≧50% of the lesion diameter or in case of relapse.

## 3. Results

The mean age at diagnosis was 13.8 with a male prevalence (31 males, 15 females). The anatomical location, collected in Table 1, shows a predominant distribution in the proximal metaphysis of long bones and in the pelvis. The cysts were classified in inactive, active, and aggressive according to the Enneking staging of musculoskeletal tumours as shown in Table 2: they were all active, except for one that was an aggressive lesion. According to the Capanna staging (Table 3), all patients had type II lesions, i.e., central and affecting the entire diameter of the bone, except for two cases having eccentric lesion, defined as type III. Thirty-six patients healed after the first treatment; among them, four had an open curettage and the cavity was filled with bone chips and autologous BMC. Of the remaining ten patients, seven healed with 2 percutaneous injections; of the remaining two, 3 had percutaneous injection and one had a resection with total hip replacement. Healing was assessed using the modified Neer classification (Table 4) used for unicameral bone cyst treatment: at the final follow-up, thirty-six patients healed with a type II aspect, nine healed with a type I aspect, and one patient was not classified having total hip arthroplasty.

## 4. Discussion

The application of bone marrow concentrate (BMC) in the treatment of aneurysmal bone cysts (ABC) is a quite new procedure. The use of BMC to fill bony defects is well known, with a success rate that seems to be related to the number of progenitors in the graft [12]. ABC are reactive and locally aggressive lesions having osteolytic, hyperplastic, hyperemic, and hemorrhagic features; they are uncommon and rarely present after 30 years of age. Aneurysmal bone cyst-like modifications can be observed also in other pathological conditions, in malignant tumours such as telangiectatic osteosarcoma, and this eventuality must be ruled out through the histological study of specimens [6]. The aim of ABC treatment is to arrest their potentially high destructive capability. In the past years, different approaches have been proposed for ABC treatment ranging from resection to mini-invasive percutaneous procedures, and still today, there is no consensus on the best treatment [13–15]. However, the analysis of ABC pathogenesis and natural history, although not completely understood, can be helpful to chose the most appropriate treatment. It was long thought that ABC's cause was a vascular impairment due to abnormal venous circulation with osteoclast activation and local bone resorption; a more recent clonal theory overtook the vascular theory: the origin of the lesion seems to be associated with a translocation of USP6 oncogene on chromosome 17 [16, 17]. This finding confirms the oncological nature of ABC and therefore their high evolutivity: as for other musculoskeletal tumors, ABC can be classified as inactive, active, and aggressive and their evolution proceeds in phases. The initial osteolysis can evolve to cortical destruction and periosteal reaction creating a bulky bone; the appearance of septa attests a stabilization and remodelling attempt that in a few cases can lead to spontaneous resolution. In other cases, ABC assume an aggressive and destructive behaviour; however, the reason that shifts to a stabilizing or a destructive pattern is still unknown [7, 18]. Delloye et al. were among the first to report two cases of ABC treated with BMC: the rationale underlying this treatment was in the inductive properties of mesenchymal stems cells [19]. Many studies have already confirmed that BMC, particularly, if obtained through iliac crest aspiration, is rich in mesenchymal cells that seem to represent the source of osteoblastic elements during growth, remodelling, and bone reparative processes [20–22]. Moreover, ABCs are subject to continuous reparative processes in a cavity filled with blood that provides an ideal environment for mesenchymal cells to express their inductive capacities. Therefore, the aim of our treatment was to induce spontaneous ossification of ABC through a minimally invasive procedure. Currently, there is no consensus on the ideal treatment of ABC. For eccentric and aggressive lesions, located in expendable bones like the proximal fibula or pubic ramus, many authors have advocate resection as treatment of choice, reporting very low recurrence rates [23, 24]. According to other authors, curettage with or without bone grafting seems to be the best choice, reporting acceptable recurrence rates with a much better functional outcome than resection [25, 26]. More recently, in consideration of patients' young age and with better understanding of ABC pathogenesis, less-invasive procedures are gaining more success: percutaneous embolization, isolated intracystic injection of demineralized bone powder, bone marrow, calcitonin and Ethibloc are some examples [27–29]. Docquier and Delloye reported good results inducing ABCs' healing with intralesional implantation, through a mini-invasive access, of a bone paste made of autogenic bone marrow and allogenic bone powder: the goal of this treatment was to interrupt the destructive osteoclastic process and promote spontaneous bone regeneration [7]. In our series, with the technique described, we obtained a full recovery after only one treatment in more than 50% of our cases; the remaining had a maximum of two other procedures except for one patient, affected by a lesion involving the femoral head in contact with the articular cartilage that eventually underwent prosthetic replacement. Only four patients received curettage as first treatment since they had wide lesions; they both reported a Neer II grade at follow-up. As regards percutaneous approach, we believe that, given the age of patients affected by this condition, the proposed treatment avoids extensive surgery and blood loss and can be repeatable with minor discomfort for the patient. Moreover, we did not report any complication, including superficial or deep infection, fracture, or other adverse reactions, both at the harvest site and at the application site: this confirmed the safety of the procedure used.

Nevertheless, even if the results obtained are promising, the number of cases is exiguous and a larger group is necessary to confirm the validity of this procedure; perhaps with a greater number of patients a stratification based on anatomical location and cyst dimension would be feasible and would help in the choice of the best treatment.

## 5. Conclusions

Even if affected by the limitation of a relatively small number of patients, our case series proves that bone marrow concentrate is easy to obtain and to manipulate, and can be immediately available in a clinical setting; it offered good results with no complications. We believe that cavitary lesions like ABCs are ideal settings for using mesenchymal cells since they act as a biological chamber where the healing processes can be potentiated by the presence of blood and growth factors that stimulate differentiation of an osteogenic lineage.

## Figures and Tables

**Table 1:** ABC anatomical location.

Location	
Proximal humerus	14
Humeral diaphysis	8
Pelvis	3
Femur diaphysis	6
Proximal femur	13
Calcaneus	2

**Table 2:** Enneking classification.

Inactive	Intact, well-defined margins
Active	Incomplete margins but well-defined lesion
Aggressive	Poorly defined margins with reactive bone formation

**Table 3:** Capanna classification.

Type	Morphological features
I	Central lesion
II	Central lesion involving the entire bone diameter
III	Eccentric lesion
IV	Subperiosteal lesion
V	Subperiosteal lesion extending to soft tissues

**Table 4:** Modified Neer classification.

TYPE	Description
I	Cyst healed with radiolucent area < 1 cm
II	Cyst healed with radiolucent area < 50% of diameter and enough cortical thickness
III	Persistent cyst with radiolucent area > 50% of diameter with thin cortical rim
IV	Recurrent cysts in the obliterated area or increased residual radiolucent area

## Data Availability

The data sets used and/or analysed during the current study are available from the corresponding author on reasonable request.

## References

[1] Friedenstein A. J., Piatetzky S., Petrakova K. V. (1966). Osteogenesis in transplants of bone marrow cells. *Journal of Embryology and Experimental Morphology*.

[2] Friedenstein A. J., Chailakhjan R. K., Lalykina K. S. (1970). The development of fibroblast colonies in monolayer cultures of guinea-pig bone marrow and spleen cells. *Cell and Tissue Kinetics*.

[3] Owen M., Friedenstein A. J. (1988). Stromal stem cells: marrow-derived osteogenic precursors. *Ciba Foundation Symposium*.

[4] De Biase P., Campanacci D. A., Beltrami G. (2011). Scaffolds combined with stem cells and growth factors in healing of pseudotumoral lesions of bone. *International Journal of Immunopathology and Pharmacology*.

[5] Jäger M., Herten M., Fochtmann U. (2011). Bridging the gap: bone marrow aspiration concentrate reduces autologous bone grafting in osseous defects. *Journal of Orthopaedic Research*.

[6] Campanacci M. (2013). *Bone and Soft Tissue Tumors*.

[7] Docquier P. L., Delloye C. (2005). Treatment of aneurysmal bone cysts by introduction of demineralized bone and autogenous bone marrow. *The Journal of Bone and Joint Surgery American Volume*.

[8] Cottalorda J., Bourelle S. (2006). Current treatments of primary aneurysmal bone cysts. *Journal of Pediatric Orthopaedics. Part B*.

[9] Enneking W. F., Spanier S. S., Goodman M. A. (1980). A system for the surgical staging of musculoskeletal sarcoma. *Clinical Orthopaedics and Related Research*.

[10] Capanna R., Bettelli G., Biagini R., Ruggieri P., Bertoni F., Campanacci M. (1985). Aneurysmal cysts of long bones. *Italian Journal of Orthopaedics and Traumatology*.

[11] Neer C. S., Francis K. C., Marcove R. C., Terz J., Carbonara P. N. (1966). Treatment of unicameral bone cyst. A follow-up study of one hundred seventy-five cases. *The Journal of Bone and Joint Surgery American Volume*.

[12] Hernigou P., Poignard A., Beaujean F., Rouard H. (2005). Percutaneous autologous bone-marrow grafting for nonunions influence of the number and concentration of progenitor cells. *The Journal of Bone and Joint Surgery. American Volume*.

[13] Flont P., Kolacinska-Flont M., Niedzielski K. (2013). A comparison of cyst wall curettage and en bloc excision in the treatment of aneurysmal bone cysts. *World Journal of Surgical Oncology*.

[14] Abuhassan F. O., Shannak A. O. (2009). Subperiosteal resection of aneurysmal bone cysts of the distal fibula. *Journal of Bone and Joint Surgery. British Volume (London)*.

[15] Garg N. K., Carty H., Walsh H. P., Dorgan J. C., Bruce C. E. (2000). Percutaneous Ethibloc injection in aneurysmal bone cysts. *Skeletal Radiology*.

[16] Nielsen G. P., Fletcher J. A., Oliveira A. M., Fletcher B. J. A., Hogendoorn P. C. W., Mertens F. (2013). Aneurysmal bone cyst. *WHO Classification of Tumours of Softtissue and Bone*.

[17] Ye Y., Pringle L. M., Lau A. W. (2010). TRE17/USP6 oncogene translocated in aneurysmal bone cyst induces matrix metalloproteinase production via activation of NF-*κ*B. *Oncogene*.

[18] Malghem J., Maldague B., Esselinckx W., De Nayer P., Vincent A. (1989). Spontaneous healing of aneurysmal bone cysts. A report of three cases. *The Journal of Bone and Joint Surgery. British volume*.

[19] Delloye C., De Nayer P., Malghem J., Noel H. (1996). Induced healing of aneurysmal bone cysts by demineralized bone particles. A report of two cases. *Archives of Orthopaedic and Trauma Surgery*.

[20] Bruder S. P., Fink D. J., Caplan A. I. (1994). Mesenchymal stem cells in bone development, bone repair, and skeletal regenaration therapy. *Journal of Cellular Biochemistry*.

[21] Owen M., Peck W. A. (1985). Lineage of osteogenic cells and their relationship to the stromal system. *Bone and Mineral*.

[22] Oreffo R. O., Triffitt J. T. (1999). Future potentials for using osteogenic stem cells and biomaterials in orthopedics. *Bone*.

[23] Campanacci M., Capanna R., Picci P. (1986). Unicameral and aneurysmal bone cysts. *Clinical Orthopaedics*.

[24] Cottalorda J., Kohler R., Lorge F. (2004). Aggressive aneurysmal bone cyst of the humerus in a child. *Revue de Chirurgie Orthopédique et Réparatrice de l'Appareil Moteur*.

[25] Vergel de Dios A. M., Bond J. R., Schives T. C., McLeod R. A., Unni K. K. (1992). Aneurysmal bone cyst. A clinicopathologic study of 238 cases. *Cancer*.

[26] Tillmann B. P., Dahlin D. C., Lipscomb P. R., Steewaart J. R. (1968). Aneurysmal bone cysts, analysis of 95 cases. *Mayo Clinic Proceedings*.

[27] Cottalorda J., Bourelle S. (2007). Aneurysmal bone cyst in 2006. *Revue de Chirurgie Orthopédique et Réparatrice de l'Appareil Moteur*.

[28] Amendola L., Simonetti L., Simoes C. E., Bandiera S., de Iure F., Boriani S. (2013). Aneurysmal bone cyst of the mobile spine: the therapeutic role of embolization. *European Spine Journal*.

[29] Adamsbaum C., Mascard E., Guinebretière J. M., Kalifa G., Dubousset J. (2003). Intralesional Ethibloc injections in primary aneurysmal bone cysts: an efficient and safe treatment. *Skeletal Radiology*.

